# Storylines of family medicine III: core principles—primary care, systems and family

**DOI:** 10.1136/fmch-2024-002790

**Published:** 2024-04-12

**Authors:** William B Ventres, Leslie A Stone, Timothy A Joslin, John W Saultz, Sommer Aldulaimi, Paul R Gordon, John C Lane, Eric R Lee, Jacob Prunuske, Limor Gildenblatt, Michael H Friedman, Colleen T Fogarty, Susan H McDaniel, Tessa Rohrberg, Amy Odom

**Affiliations:** 1 Family and Preventive Medicine, University of Arkansas for Medical Sciences College of Medicine, Little Rock, Arkansas, USA; 2 Family Medicine, Oregon Health & Science University, Portland, Oregon, USA; 3 Family and Community Medicine, University of Arizona Medical Center–South Campus, Tucson, Arizona, USA; 4 Family and Community Medicine, University of Arizona Medical Center–University Campus, Tucson, Arizona, USA; 5 UAMS West Regional Campus Family Medicine Residency Program, Fort Smith, Arkansas, USA; 6 Medical College of Wisconsin–Central Wisconsin Campus, Wausau, Wisconsin, USA; 7 Family and Community Medicine, Medical College of Wisconsin–Central Wisconsin Campus, Wausau, Wisconsin, USA; 8 Family Medicine, Advocate Illinois Masonic Medical Center, Chicago, Illinois, USA; 9 Family Medicine, Presence Saints Mary and Elizabeth Medical Center, Chicago, Illinois, USA; 10 Family Medicine, University of Rochester Medical Center, Rochester, New York, USA; 11 Psychiatry and Family Medicine, University of Rochester Medical Center, Rochester, New York, USA; 12 Family and Community Medicine, University of Kansas School of Medicine-Wichita, Wichita, Kansas, USA; 13 Sparrow/Michigan State University Family Medicine Residency Program, Lansing, Michigan, USA

**Keywords:** Family Medicine, General Practice, Primary Health Care, Professional-Family Relations, Physician-Patient Relations

## Abstract

*Storylines of Family Medicine* is a 12-part series of thematically linked mini-essays with accompanying illustrations that explore the many dimensions of family medicine, as interpreted by individual family physicians and medical educators in the USA and elsewhere around the world. In ‘III: core principles—primary care, systems, and family’, authors address the following themes: ‘Continuity of care—building therapeutic relationships over time’, ‘Comprehensiveness—combining breadth and depth of scope’, ‘Coordination of care—managing multiple realities’, ‘Access to care—intersectional, systemic, and personal’, ‘Systems theory—a core value in patient-centered care’, ‘Family-oriented practice—supporting patients’ health and well-being’, ‘Family physician as family member’ and ‘Family in the exam room’. May readers develop new understandings from these essays.

## Introduction

Family medicine is one of the primary care disciplines of medicine. As such, it is founded on several principles of primary care, each of which is supported by research that demonstrates its importance to a healthcare system. In fact, systems thinking is one of the major factors that distinguish family medicine from other primary care disciplines. Wise family physicians integrate systems into their diagnostic and therapeutic activities. They balance their understanding of patients with recognition of the fact that we all live and work in a complex, interconnected world. In sum, many factors influence the health and well-being of patients, among them patients’ families of origin and chosen families.

## Continuity of care—building therapeutic relationships over time

Tim Joslin and John Saultz

*Continuity is one of the core values of family medicine. Although continuity has multiple dimensions, the ongoing interpersonal doctor–patient relationship, which continues across a lifespan, is the most defining characteristic of family medicine.*

Long-term relationships are what allow family physicians *entrée* into their patients’ lives. Family physicians care for people over the course of years—from cradle to grave, as individuals and as members of multigenerational family groups.^1^

Bernice—a single name will suffice. She is a patient in our practice that everyone knows. When I (TAJ) met her halfway through my family medicine residency, she had already experienced a handful of primary care clinicians over the preceding dozen years. Typically, a resident would assume her care at the beginning of training and pass her on to another at graduation. Consistency and continuity lasted for only three years at a time. When I inherited Bernice, the story could have been the same, but something different happened. I stuck around, and so did she. After graduating residency, I stayed on as the chief resident and later as a faculty member.

The day I met Bernice, the medical assistant (MA) nudged me just before I entered the examination room. I looked back at her, a puzzled expression on my face.

*What was that for?* I wondered.

’You’ll see,’ the MA said just before I entered the room.

As I entered, I saw Bernice in her typical state, conditioned by her persistent mental illness. Her well-worn jacket was no longer waterproof, perforated by cigarette burns. Her sweats were dirty, and she wore sandals over a set of wool socks. Her hair was thin, dark and unkempt, and her fingers were a dark burnt caramel colour—she smoked nearly 100 self-rolled cigarettes per day. I noticed her eyes scanning the room, as if she were trying to physically escape her racing thoughts.

Like most second year residents, I tried to wrap my mind around the basics during my first appointment with Bernice. I learnt that she had a history of multiple psychiatric hospital admissions; attempts to get her reconnected with outpatient mental health services had been invariably unsuccessful. Follow-up plans never came to fruition. Her adherence to medication therapy was sporadic, at best.

There was no magical connection in our relationship at the beginning, but over weeks, months and years, a connection formed between us. Mutual trust ensued. Through our team’s intense, coordinated and prolonged efforts, Bernice’s life became less chaotic. Her hospital admissions decreased drastically. She often went years between psychiatric hospitalisations.

Bernice’s life will never be easy, but the relationship that formed and grew over many years resulted in meaningful improvements in her health and day-to-day existence.

The interpersonal domain of continuity, characterised by personal trust, professional intimacy and mutual responsibility, is hard to teach—medical students on their clerkships rarely see people more than once, and family medicine residents commonly spend significant periods away from ambulatory care. Yet, continuous physician–patient relationships are what many family physicians find most satisfying in their careers.

**Figure 1:**
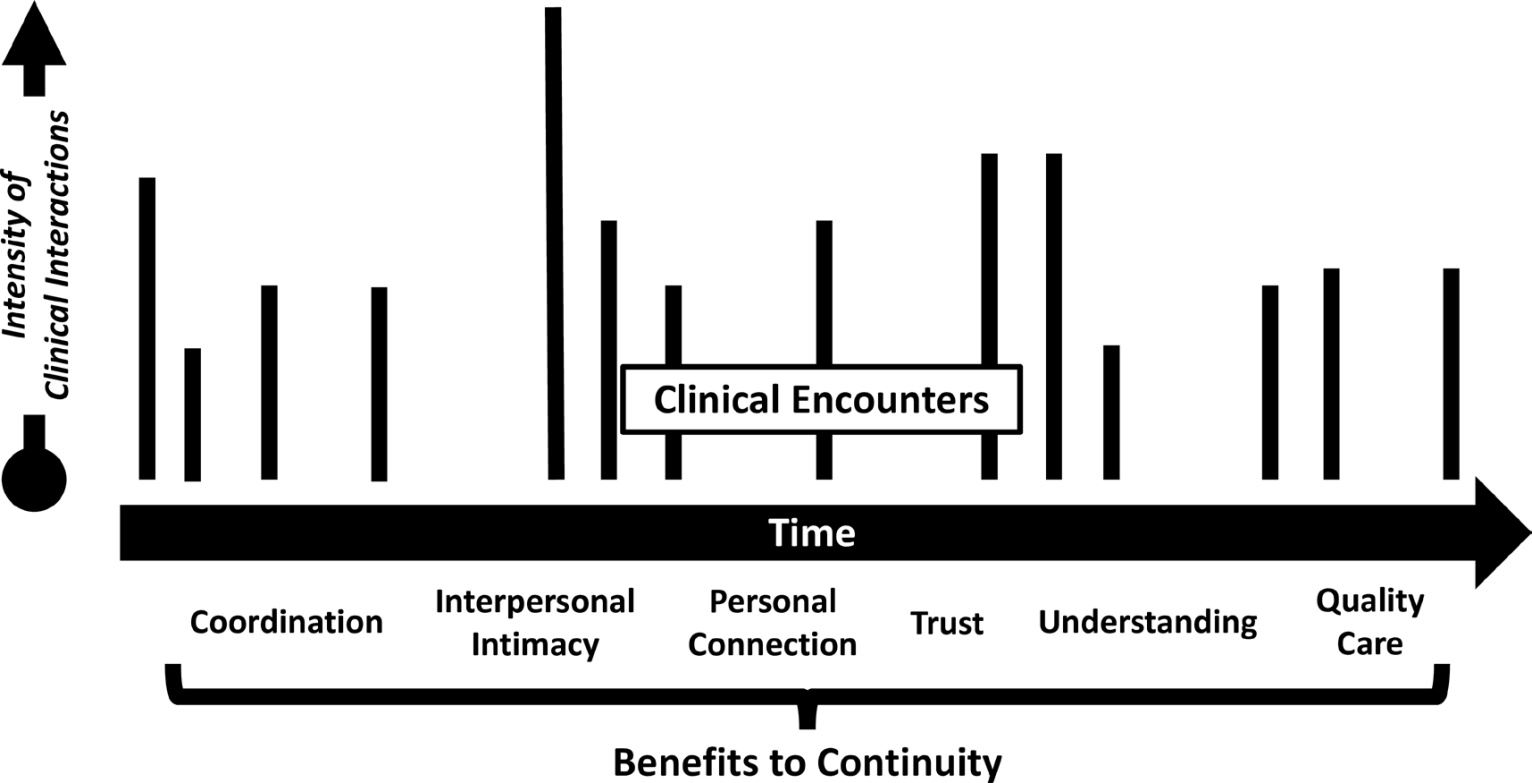
Tracking continuity of care: time versus intensity.

Improved continuity of care lowers healthcare costs, reduces risk of hospitalisation, improves patient and clinician satisfaction and improves overall quality of care.^2–4^ Continuity nourishes the seed that is planted when doctors and patients first meet, allowing healing relationships to grow into something wonderful and unexpected (figure 1).

### Readings

- Loxterkamp D. The lost pillar: does continuity of care still matter? *Ann Fam Med* 2021;19:553–5. doi: 10.1370/afm.2736
- Nowak DA, Sheikhan NY, Naidu SC, Kuluski K, Upshur REG. Why does continuity of care with family doctors matter? Review and qualitative synthesis of patient and physician perspectives. *Can Fam Physician* 2021;67:679–88. doi: 10.46747/cfp.6709679
- Saultz JW. Defining and measuring interpersonal continuity of care. *Ann Fam Med* 2003;1:134–43. doi: 10.1370/afm.23

## Comprehensiveness—combining breadth and depth of scope

Sommer Aldulaimi and Paul Gordon

*Family physicians are trained to see and manage a variety of different medical illnesses and conditions over the lifetimes of patients in a variety of settings. This comprehensiveness of care is key to improving care, decreasing healthcare costs and working towards health equity.*

**Figure 2:**
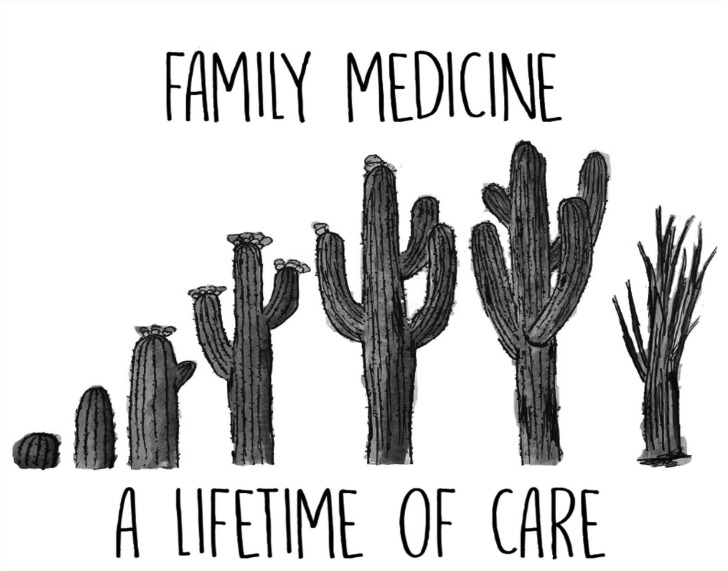
Comprehensiveness across the lifespan. Adapted with permission.^7^

In the same morning, a family physician might see a two year-old for a well-child check; attend to an elderly male for his diabetes, high blood pressure and heart failure; do a Pap smear on a young woman as part of a well-woman examination and discuss birth control options; inject a patient’s knee; treat a urinary tract infection; and deliver a baby. Family physicians are trained to see and manage a variety of different medical illnesses and conditions in a variety of clinical settings; they must manage these illnesses and conditions over the lifetimes of their patients regardless of sex or presenting condition.^5 6^ Such breadth and depth of scope by which family physicians attend to patients’ medical problems is called comprehensiveness (figure 2).^5 7^

Comprehensiveness is one of the core principles of primary care,^6 8^ though family physicians typically have a scope of practice that is broader than other primary care specialties. Such scope often includes practising in inpatient, outpatient and community settings; attending to maternity, paediatric, adult and elderly patients; and offering services that include procedures, minor surgeries and public health leadership.^6^

Why should family physicians and patients care about comprehensive practice?

Comprehensiveness is associated with numerous benefits. These include greater efficiency (family physicians can address most patients’ needs without the need for subspecialty consultation) with better health outcomes provided at lower costs. Comprehensiveness results in lower costs for patients and for the healthcare system.^5^

National healthcare systems that focus on comprehensive primary care spend much less money and have better outcomes than those systems that are focused on subspecialties.^6^ Comprehensive care is also associated with decreased rates of hospitalisations and better self-reported health outcomes by patients themselves.^5 6^ Perhaps even more importantly, in healthcare systems that emphasise comprehensive care, disparities in disease severity decrease while population-based markers of prevention increase.^5^

Comprehensiveness and broader scope of practice have been correlated with decreased rates of burnout among family physicians, an important consideration in light of the primary care physician shortage that exists in the USA and in other areas around the world.^9^

Comprehensiveness is crucial to improving care and health outcomes, lowering costs of treatment, lessening health disparities and decreasing physician burnout. Family physicians are trained to provide the most comprehensive care of any medical specialty, regardless of geographical location, patient demographic or method of reimbursement for services rendered. Any rational healthcare system should facilitate and foster this type of comprehensiveness in medical care.

### Readings

- Cubaka VK, Dyck C, Dawe R, *et al*. A global picture of family medicine: the view from a WONCA storybooth. *BMC Fam Pract* 2019;20:129. doi: 10.1186/s12875-019-1017-5
- Grumbach K. To be or not to be comprehensive. *Ann Fam Med* 2015;13:204–5. doi: 10.1370/afm.1788
- Weidner AKH, Phillips RL Jr, Fang B, Peterson LE. Burnout and scope of practice in new family physicians. *Ann Fam Med* 2018;16:200–5. doi:10.1370/afm.2221

## Coordination of care—managing multiple realities

John Lane

*Prudent generalist practitioners know that coordination of care—addressing issues related to the process of navigating the healthcare system—is a crucial component of their clinical responsibilities.*

**Figure 3:**
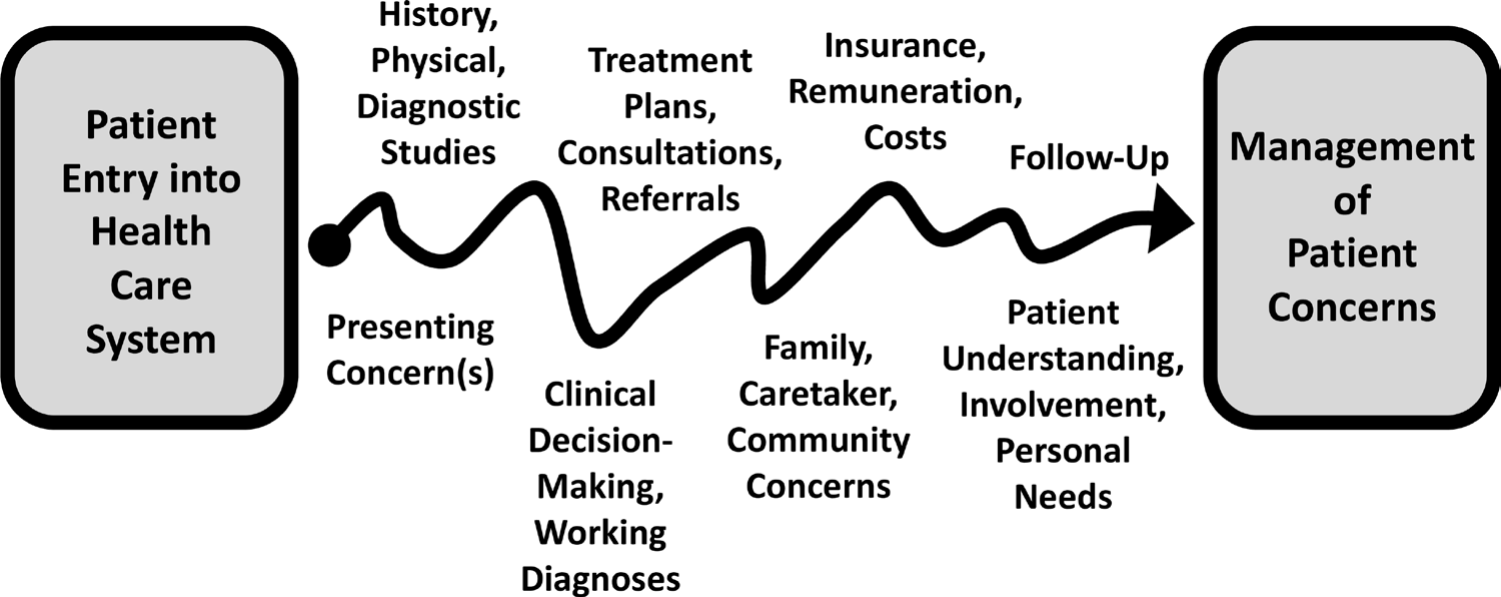
Coordination: navigating the healthcare system.

Family physicians sort symptoms and signs into orderly patterns that become diagnoses and treatment plans. Less obvious but equally important is the work of sorting complex interactions with the healthcare system into an orderly and efficient series of interventions and responses. This is called coordination of care (figure 3).

Coordination of care is a core task for all family physicians. Along with other clinical team members, they collect vital signs, review medical records, elicit clinical histories and perform pertinent physical examinations. They order laboratory tests and diagnostic studies. They arrange subspecialty consultations. They gather and synthesise data, develop therapeutic plans, prescribe treatments and recommend appropriate follow-up.

In popular imagination, the archetypal family doctor manages these tasks under one roof with minimal complexity. The physician listens to the patient’s chief complaint—let’s say, a sore throat and no cough in an otherwise healthy second grader. On examination, the child has a temperature of 102°F, inflamed tonsils with exudates and anterior cervical adenopathy. The diagnosis is conveyed, questions are answered and the child’s mother receives an antibiotic prescription. She pays the bill at the front desk, and, with her child, heads to the pharmacy. Cure is on the way.

Although such to-the-diagnostic-point encounters still exist, most clinical interactions are much more complex. They often involve the following:

- A chart review that includes many documents from a variety of sources, and often decisions need to be made about information not yet in hand.
- Decision-making about diagnostic investigations, which is influenced by factors such as availability, cost, insurance and timeliness. As well, orders must be transmitted effectively and efficiently.
- Patient involvement and needs, including social determinants of health: are there concerns beyond the clinical presentation that may affect clinical attendance, engagement with the therapeutic plan and efficacy of treatments?
- Referrals and consultations, the arrangements for which often require considerable thought and logistical skills to complete.

Consider, then, a more complex case. A family physician sees a patient in follow-up for abdominal pain. The history and physical examination suggest a wide differential diagnosis. Some pertinent imaging results are available from a recent emergency department (ED) visit but not the laboratory results or ED physicians’ impressions. Furthermore, there was a distant, similar medical event in which the patient had an extensive workup and diagnosis, but the findings and impression are presently obscure.

The family physician considers a variety of questions. How quickly can previous results be received? At what expense, risk and patient distress can new testing and consultations be obtained? Can the patient attend an important family event before testing and consultations are completed? Can the clinic’s team facilitate the patient’s journey when the above questions have been answered?

Successful coordination of care depends on a high-functioning team with sophisticated systems to manage the to-and-from flow of information pertinent to patient care. Such success depends on strategic planning, respectful communication, consistent work habits and people—team members—who understand the patient experience, are forward thinking and practise resiliency daily.

This is coordination of care. Done poorly, patients suffer. Done well, it enhances the experience of all participants in the healthcare system.

### Readings

- Bodenheimer T. Coordinating care—a perilous journey through the health care system. *N Engl J Med* 2008;358:1064–71. doi: 10.1056/NEJMhpr0706165
- Bodenheimer T, Ghorob A, Willard-Grace R, Grumbach K. The 10 building blocks of high-performing primary care. *Ann Fam Med* 2014;12:166–71. doi: 10.1370/afm.1616
- Phillips C. Care coordination for primary care practice. *J Am Board Fam Med* 2016;29:649–51. doi: 10.3122/jabfm.2016.06.160312

## Access to care—intersectional, systemic and personal

Eric Lee and Jake Prunuske

*Access to healthcare is essential for optimal health. Family physicians improve access to care by offering community-based, continuity-oriented medical services in the context of individuals’ values, family systems and communities.*

Healthcare access is ‘the possibility to identify healthcare needs, to seek services, to reach resources, to obtain or use healthcare services, and to be offered services appropriate to the needs for care.’^10^ Ideally, the resources of clinicians, health systems and communities meet the needs and align with the abilities of patients. Optimal access requires health literacy, reciprocal trust between clinicians and patients, respect for and integration of personal and cultural values, geographical proximity, a supportive infrastructure and affordable costs. Failure to attend to these factors has led to limited access to healthcare for many people in the USA, where unequal access to healthcare is a major challenge. A strong system of primary care is necessary to ensure that high-quality care is available to every individual and family in every community.^11^

Importantly, people must first trust that the doctors who attend to them will understand their needs and perspectives and will work in their best interest. Unfortunately, issues in the USA (such as inequity, racism and biases) have contributed to a culture of distrust. One way to rebuild trust is to improve the diversity of the US healthcare workforce.^12^ Relative to other specialties, family medicine has higher racial and ethnic representation and greater presence in ethnically diverse, medically underserved communities.^13^

The high cost of healthcare in the USA is another common cause of deferred care and treatment, especially for people who are medically underserved by current healthcare systems.^14^ Family doctors’ emphasis on providing a broad scope of care and following principles of continuity, context and community contributes to decreased costs of care and fewer hospitalisations, emergency room visits and surgeries.^15^ Much of the cost burden faced by patients could be mitigated or even avoided if more patients regularly saw their family doctor.

Family doctors thus play a critical role in addressing multifactorial, intersectional, personal and systemic barriers to access to medical care. They are well equipped to meet the social and cultural needs of populations in their communities. At the heart of family medicine is a drive to provide whole-person care—personalisation, relationship building and continuity are core values of family medicine.^16^

The relationships that family doctors develop over years—and in some cases, decades—provide insight into the biological, psychosocial, cultural and political determinants of health that affect their patients. Such breadth of knowledge helps family doctors coordinate care and advocate for patients’ health needs within a multidisciplinary care team.

**Figure 4:**
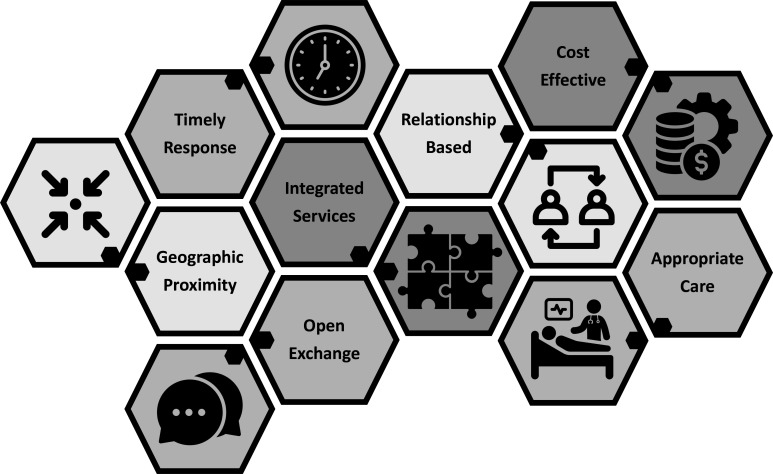
Access to healthcare: supportive factors.

The health of individual patients is, to a certain degree, dependent on access to high-quality healthcare. Along with other community-based generalist clinicians, family physicians help ensure this access. Access to care is vital to improving the overall health of people within communities, and family physicians play an integral part in providing this access (figure 4)!

### Readings

- Levesque JF, Harris MF, Russell G. Patient-centred access to health care: conceptualising access at the interface of health systems and populations. *Int J Equity Health* 2013;12:18. doi: 10.1186/1475-9276-12-18
- National Academies of Sciences, Engineering, and Medicine; Health and Medicine Division; Board on Health Care Services; Committee on Implementing High-Quality Primary Care. *Implementing High-Quality Primary Care: Rebuilding the Foundation of Health Care*. Consensus Study Report/Highlights. May 2021. Available: https://nap.nationalacademies.org/resource/25983/Highlights_High-Quality/Primary/Care-4.23.21_final.pdf [Accessed 31 Jan 2024].
- Nowak DA, Sheikhan NY, Naidu SC, Kuluski K, Upshur REG. Why does continuity of care with family doctors matter? Review and qualitative synthesis of patient and physician perspectives. *Can Fam Physician* 2021;67:679–88. doi: 10.46747/cfp.6709679

## Systems theory—a core value in patient-centred care

Limor Gildenblatt and Mike Friedman

*Systems theory posits that complicated interaction of unique, inter-related components determines outcomes in a variety of realms. Family medicine has embraced systems theory as a core of the specialty and in so doing has given rise to a new understanding of patient-centred care.*

General systems theory emerged in the late 1940s and early 1950s to better understand the interactions between individual components of complex problems.^17^ Initially targeted to disciplines such as math, science and engineering, systems thinking has come to exert a strong influence on medical education.^18^

**Figure 5:**
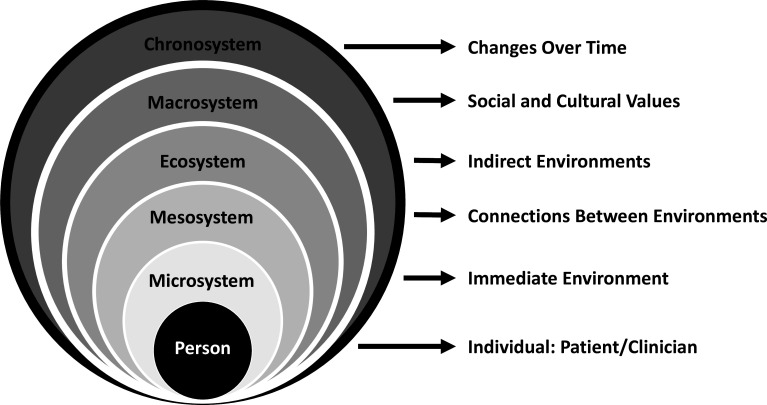
Systems theory: a graphic overview. Adapted with permission.^20^

But that wasn’t always the case. Early medical educators focused narrowly on training students to master the biomedical basis of disease—many still do! This reductionist model derived from the notion that breakdowns in biological processes could fully explain disease and illness; thus, if physicians could reduce problems into discrete, quantifiable units, they could better understand the whole. In time, physicians in all fields saw the limitations of this simple biomedical model. What was missing was context and an appreciation of the complex interactions that exist in our patients’ worlds (figure 5).^19 20^

Consider the following case: Rosa, a 59-year-old second-generation immigrant from Mexico, was diagnosed with new-onset diabetes and hypertension. Despite appropriate referrals and educational interventions, her problems remained uncontrolled. She worked long hours at an unfulfilling desk job that she didn’t dare leave because she didn’t want to risk her pension or insurance.

When asked if her schedule could be modified to accommodate her health needs, Rosa revealed the factors that made such adjustments impossible: her husband didn’t work because of his own health problems; her youngest child, aged 25, couldn’t hold a job; the oldest son, in his 30s, also lived with the family, along with his partner and two young children. Finally, her middle son, separated from his wife, had been driving with their two children when he ran into a highway overpass, instantly killing one of the children. Owing to a greatly elevated blood alcohol level, he was found guilty of involuntary manslaughter and was sentenced to eight years in prison. But his estranged wife and the surviving child moved into the house as well.

The biomedical model of healthcare was clearly incapable of capturing her situation. Her diabetes and hypertension had social and cultural influences just as surely as they had a biological component.

In the relatively young field of family medicine, where managing individual patients in the context of family and community has always been a core principle, the need to develop an expansive view of systems-based care quickly became evident. Leaders in the discipline embarked on a national strategy to introduce a broader perspective to patient care. They didn’t reject biology but instead incorporated the previously neglected non-biological phenomena into patient care.

This approach, which presaged what has come to be known as holistic care, employed the biopsychosocial model of health.^21 22^ It accepted that human pathology arose not simply from failures in biological pathways but was impacted by a complex network of inter-related factors. Applying an understanding of these disparate psychological, social and environmental components not only offered patients a clearer path to health; it also gave physicians the opportunity to better manage their own expectations, work collaboratively with other healthcare professionals and truly provide patient-centred care.

### Readings

- Card AJ. The biopsychosociotechnical model: a systems-based framework for human-centered health improvement. *Health Syst (Basingstoke)* 2022;12:387-407. doi: 10.1080/20476965.2022.2029584
- Sturmberg JP. Systems and complexity thinking in general practice: part 1 - clinical application. *Aust Fam Physician* 2007;36:170–3.
- Sturmberg JP, Martin CM, Katerndahl DA. Systems and complexity thinking in the general practice literature: an integrative, historical narrative review. *Ann Fam Med* 2014;12:66–74. doi: 10.1370/afm.1593

## Family-oriented practice—supporting patients’ health and well-being

Colleen Fogarty and Susan McDaniel

*Family-oriented practice is the application of general systems theory to families; it focuses on the relationship between significant others—their complementary interactions, shared beliefs and effects on each other’s health and behaviours.*

What do we mean by *family*? What are its characteristics?

We define family as the many types of relationships formed by strong social bonds over time—family is ‘any set of intimates with a history and a future’.^23^

As to general characteristics, families are rarely uniform in nature. They are commonly affected by cultural expectations about independent living, child rearing and kinship networks. They are often formed and reformed through coupling and uncoupling. They are routinely influenced by previous generations and change over time in response to the realities of human development, from birth through death.^24^

**Figure 6:**
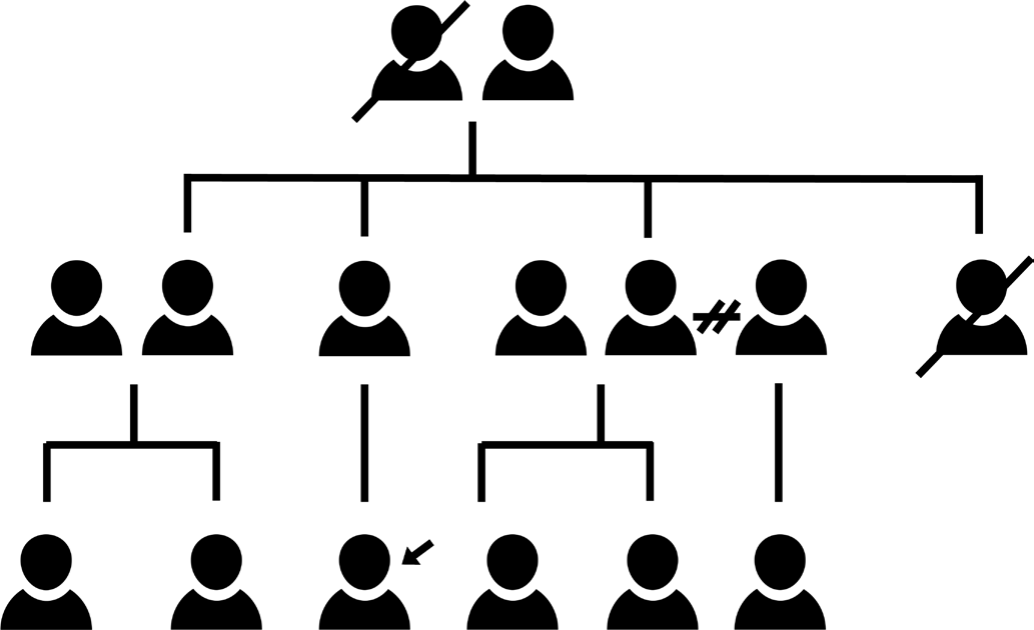
Who is in the family? A family health diagramme. 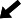
= index patient; 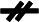
= divorce/separation; 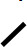 = deceased

Physicians who understand how family matters affect healing learn about their patients’ families. Using a systematic assessment informed by five key questions, family physicians work to identify family beliefs and practices that may explain patients’ symptoms. Identifying these beliefs and practices—aided by family genograms (figure 6)—also helps determine helpful resources for treatment planning.

The five questions key to family-oriented practice are as follows^25^:

1. *Does anyone in the family have the same problem as you do?*

Knowing that a condition ‘runs in the family’, by whatever mechanism, helps clinicians gauge patients’ understandings of illness. Hearing, ‘Yeah, my uncle lost his leg from diabetes,’ functions as a learning moment to assess a patient’s concern about avoiding diabetic complications.

1. *What do family members think is causing your symptom?*

Family health beliefs are shared understandings about the causes and treatments of an illness. For example, families may approach treatment of the common cold differently. Some caregivers may make chicken soup as a home remedy for a child with the common cold; others may quickly take a child to the doctor at the first sign of sniffles.

1. *Who in the family is most concerned about your problem?*

If an adult patient appears blasé about a condition, wondering aloud who else might be concerned can reveal a spouse in the waiting room or a worried child across the country. This knowledge can assist in identifying the ‘real’ reason for a visit.

1. *Have there been other changes or stressors related to your presenting concerns?*

Recognising the role of contextual factors—think stress related to a new baby, move, divorce, job loss or death in the family—helps clinicians understand their patients’ perspectives. An example: Mr Morrison has worsening chronic renal failure. During a routine visit, he mentions that his wife and he always argue about who is to do yard work. She hears her husband as critical of her abilities; he, now unable to do basic physical tasks, wants her to know what to do after he dies. Such misunderstandings are common.

1. *Who in the family can help you with your health problem?*

Patients regularly struggle with recommendations for change. Encouraging them to bring significant family members to office visits helps promote adherence to treatment plans.

Even when patients come alone to consultations, they bring with them a family system rich with shared understandings about symptoms, the meaning of illness, when to consult with professionals and how to treat problems. Physicians who learn about families can leverage these natural support systems in support of patients’ health and well-being.

### Readings

- Felix D, Mauksch L. Improving family interviewing skills using the family centered observation form: an online training module for healthcare providers. 2017. Available: http://www.fcof.us [Accessed 31 Jan 2024 ].
- Medalie JH, Cole-Kelly K. The clinical importance of defining family. *Am Fam Physician* 2002;65:1277–9.
- Waters I, Watson W, Wetzel W. Genograms. Practical tools for family physicians. *Can Fam Physician* 1994;40:282–7.

## Family physician as family member

Tessa Rohrberg

*Providing healing care within the intimacy of the doctor–patient relationship defines the family physician as a family member.*

**Figure 7:**
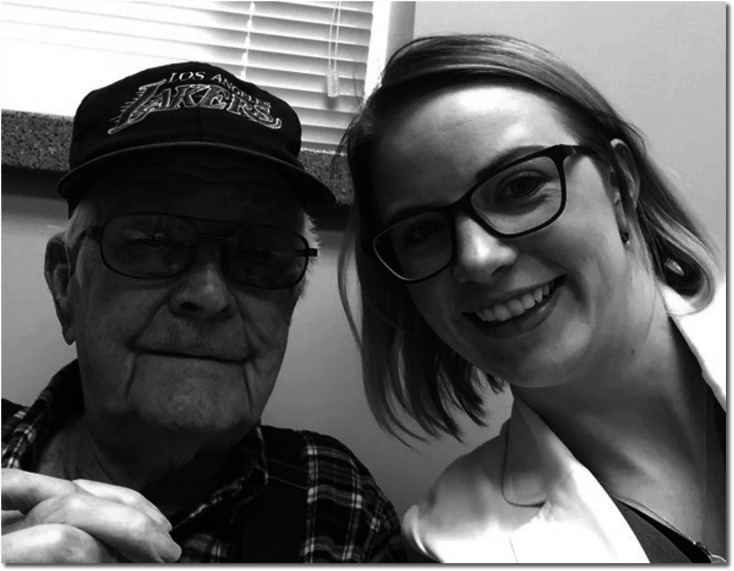
Arthur and me. Reproduced with permission.^26^

As I sit in my office and reflect on what ‘family physician as family member’ means, my eye catches a photo on my bulletin board (figure 7).^26^ It is of Arthur and me. I was new to private practice. Arthur was a World War II vet who routinely drove 30 miles each way to see me for his care.

What Arthur wanted was not prescriptions or expensive workups to investigate his chronic cough. Rather, he wanted me to know about him, and he wanted to know about me. I learnt about his passion for farming and shared that my dad owned a tractor implements business. While our visits entailed discussions of changes to his cough, they mostly focused on how his crops were doing. One day, I wheeled next to Arthur and snapped a selfie of us—Arthur in his overalls and baseball cap and I in my newly minted white coat.

Of course, we as family physicians rely on our medical knowledge to diagnose and treat patients. We have arguably the broadest of medical knowledge given the variety and complexity of the patients we care for. However, beyond the skill of diagnosing and treating disease, we characterise our specialty by the long-term relationships we have with individual patients.^27^

While family physicians often care for generations of families and are trained to appreciate family as a social construct within community, family medicine is not named solely for care of the family unit. Lynn Carmichael, a founder of the Society of Teachers of Family Medicine, maintained that family medicine focused on a close familiarity borne of caring for the whole patient rather than the family as a clinical construct.^28^ This familiarity emerged from four components: affinity, continuity, intimacy and reciprocity.^29^

As with all human relationships, the doctor–patient relationship is susceptible to ups and downs filled with positive and negative emotions.^27^ It is through this reciprocal relationship that people’s humanity is revealed; it is this relationship that brings value to both patients and family physicians. Together, we celebrate successes and grieve losses.

We, as family physicians, provide a shoulder for patients to lean on. We work into the late hours reading and learning to aid in diagnosis. We coordinate care. We disagree. We admit mistakes. We listen and forgive. We develop a closeness that can only be described as family—part of ‘any group of intimates with a history and a future’.^23^

Eventually, Arthur’s cough led to pneumonia, and he ended up in the hospital. Examinations revealed metastatic cancer. In accordance with his wishes, I signed him up for hospice. Thereafter, it was I who drove the 30 miles to visit Arthur at his home. Beside his bed were pictures of his wife and children, a letter of recognition as a veteran and our photo. I saw it again one more time at his funeral service.

Looking at this photo now, I am reminded of my cherished connections with patients. I realise they represent what *family physician as family member* means. Medicine is my profession, but the enduring relationships built through providing holistic care are what I hold closest to heart in the work I do.

### Readings

- McWhinney IR. William Pickles Lecture 1996. The importance of being different. *Br J Gen Pract* 1996;46:433–6.
- Carmichael LP. Forty families—A search for the family in family medicine. *Fam Syst Med* 1983;1:12–6.
- Carmichael L. Voices from family medicine: Lynn Carmichael. Interview by William B. Ventres and John J. Frey. *Fam Med* 1992;24:53–7.

## Family in the examination room

Amy Odom

*Both patients and physicians are influenced by their family experiences as they interact in the examination room.*

How often do patients bring their families with them into the examination room? How often do you take your family into the examination room with you? Though it is easy to answer the first question with sometimes, and the latter with never, the answer to both questions is *always*.

Individual patients represent a collective make-up of past experiences and family influences. Similarly, our own values and perspectives have developed through our personal and family experiences. Having a family-oriented approach to patient care means considering how patients’ family contexts influence their reasons for seeking medical care and understandings of medical issues.^25^ ^30^

The importance of this cannot be underestimated. For instance, in spousal relationships, significant others’ worries often prompt appointments; however, the patients themselves may not be similarly alarmed. This can lead to apparent non-adherence.

For example, I saw a patient because his wife noted a suspicious lesion on his back. Although the patient was not at all troubled by it, the lesion’s irregularity warranted a biopsy. Then, the patient did not show up for the procedure. At a subsequent visit, the wife (who was also my patient) complained to me in frustration about her husband’s lack of concern. Her father had died from malignant melanoma, and she simply needed the security of the biopsy for reassurance. Knowing this, I was able to have a different conversation with her husband. He proceeded with a biopsy, which thankfully was benign.

As family physicians, we are often involved in clinical situations that elicit memories or feelings from past and current family experiences. Our ability to pay attention to these emergent concerns—to be mindfully aware^31^—can enhance our empathy and aid us in setting boundaries.

Bill is a 40-year-old patient of mine who is currently unemployed and smokes cigarettes. He is overweight and struggling to get control of his diabetes. The specific details of his presentation seem straightforward. Yet, in listening to his story, I am reminded of my father, who navigated a world of unemployment, obesity and chronic disease. I catch myself wondering whether Bill also uses alcohol to cope with his stressors.

It is not wrong that I think of my dad when I see Bill, for the familiarity of context helps me connect with my patient. It is important that I recognise this connection, just as it is important that I separate myself from my dad’s experience so that I may see Bill in a unique light and learn his own very personal story.

**Figure 8:**
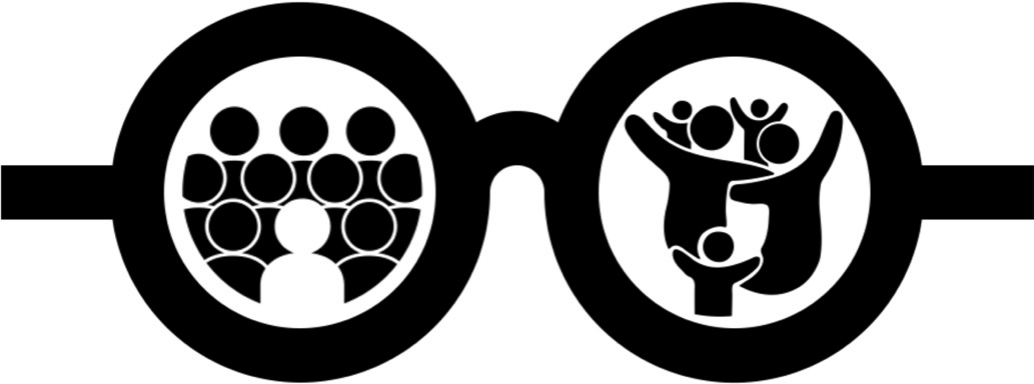
Looking through a family lens.

Family experiences inform how we and our patients approach each other; they frame the lens through which we see the world. To create strong therapeutic relationships with our patients, it is imperative we as family physicians seek to understand the influence of the family on both sides of the stethoscope (figure 8).

### Readings

- Candib LM, Savageau JA, Weinreb L, Reed G. Inquiring into our past: when the doctor is a survivor of abuse. *Fam Med* 2012;44:416-24.
- Mengel MB, Mauksch LB. Disarming the family ghost: a family of origin experience. *Fam Med* 1989;21:45-9.
- Montgomery L, Loue S, Stange KC. Linking the heart and the head: humanism and professionalism in medical education and practice. *Fam Med* 2017;49:378–83.

## Data Availability

Data sharing not applicable as no datasets generated and/or analysed for this study.
